# LASSO-Cox model in the prognostic evaluation of radiochemotherapy efficacy for lymph node metastatic nasopharyngeal carcinoma

**DOI:** 10.3389/fonc.2025.1606967

**Published:** 2025-06-04

**Authors:** Simin Lu, Xin Zhang, Xin Zheng, Gang Li, Haizhen Zhang, Yinghong Zhong, Daijie Wang

**Affiliations:** Department of Oncology, Luzhou People’s Hospital, Luzhou, China

**Keywords:** LASSO-Cox, nasopharyngeal carcinoma, radiochemotherapy, nomogram, lymph node metastatic

## Abstract

**Background:**

This retrospective study aimed to develop and validate a prognostic evaluation system based on the LASSO-Cox regression model for nasopharyngeal carcinoma (NPC) patients undergoing radiochemotherapy.

**Methods:**

Data from 186 patients treated between 2013 and 2019 at three tertiary hospitals in China were analyzed. Patients were randomly divided into a training set and a validation set in a 7:3 ratio. In the training cohort, the LASSO + Cox regression analysis was conducted to identify independent prognostic factors influencing progression-free survival (PFS). Based on these independent factors, a nomogram was constructed to predict 2-, 3-, and 5-year PFS. The predictive performance of the nomogram was then evaluated in the validation cohort.

**Results:**

Using the LASSO method for variable selection, three prognostic indicators were initially identified, and stepwise Cox regression in the training cohort further confirmed that clinical stage and EBV level were independent predictors of PFS. A nomogram was constructed based on these factors, which achieved areas under the receiver operating characteristic curves (AUC-ROC) of 0.801, 0.760, and 0.749 for predicting 2-, 3-, and 5-year PFS, respectively, in the validation cohort. The model also demonstrated robust performance through calibration and decision curve analyses.

**Conclusions:**

This nomogram provides a practical tool for personalized risk assessment and treatment planning, facilitating early identification of high-risk patients who may benefit from intensified treatment strategies.

## Introduction

Nasopharyngeal carcinoma (NPC) is a common malignancy in Southeast Asia and Southern China, often diagnosed at advanced stages with lymph node involvement, which significantly impacts prognosis. Radiochemotherapy remains the mainstay of treatment, yet outcomes vary widely due to tumor heterogeneity and individual response differences (1–3).

In recent years, advanced radiotherapy techniques, particularly intensity-modulated radiotherapy (IMRT), have revolutionized treatment by enabling more precise dose delivery that maximizes tumor control while sparing healthy tissue (4, 5). Nevertheless, individual differences in tumor radiosensitivity, radiation dosing, and fractionation schedules lead to considerable variability in treatment response and long-term survival (6). Moreover, the diversity of chemotherapy protocols further compounds the uncertainty in clinical outcomes, emphasizing the need for sophisticated prognostic models (7–9).

Traditional prognostic methods often struggle to integrate the vast array of clinical and molecular biomarkers available today, failing to fully capture their complex interactions (10). The LASSO method has emerged as a powerful tool in high-dimensional data analysis due to its ability to efficiently select the most relevant variables and reduce noise (11). When combined with the Cox proportional hazards model, the resulting LASSO-Cox framework not only accurately identifies key prognostic indicators but also provides a robust estimation of patient survival risks (12, 13). This dual advantage forms a solid foundation for tailoring personalized treatment strategies.

This study aims to develop and validate a prognostic evaluation system based on the LASSO-Cox model to predict treatment response and survival outcomes in patients with lymph node metastatic NPC undergoing radiochemotherapy. By systematically analyzing extensive clinical data and molecular markers, the research seeks to uncover the core factors influencing treatment efficacy, refine risk stratification, and ultimately support the implementation of precision medicine in NPC management.

## Materials and methods

### Study design and patient selection

This was a multi-center retrospective cohort study conducted at three tertiary hospitals in China. Data were collected from electronic medical records between January 2013 and December 2019. The study was approved by the institutional ethics committee of Luzhou People’s Hospital (Approval No. 22w202501003).

Inclusion criteria were (1): a pathological diagnosis of NPC (2); confirmed evidence of lymph node metastasis (3); treatment with a standard radiochemotherapy regimen; and (4) availability of complete clinical records and follow-up data. Patients who had received any previous systemic treatments or who had severe comorbid conditions that could affect prognosis were excluded.

### Data collection

The variables gathered included basic demographic information (such as age and gender), TNM staging, dosages of radiotherapy and chemotherapy, quantitative measurements of EBV, and follow-up data on survival and recurrence. All data were meticulously reviewed and standardized to ensure their accuracy and integrity.

### Statistical analysis

Categorical variables were analyzed using the chi-square test, while continuous variables were compared using t-tests. Categorical variables were analyzed using the chi-square test, while continuous variables were compared using t-tests. The Least LASSO regression was applied to a high-dimensional dataset comprising age, sex, T, N, stage and EBV DNA level to identify the most relevant prognostic predictors. The optimal penalty parameter (lambda) was determined using 10-fold cross-validation, aiming to minimize the mean cross-validated partial likelihood deviance. Patients were randomly divided into a training set (n = 126) and a validation set (n = 60) at a 7:3 ratio. Within the training cohort, a stepwise Cox regression analysis was performed to determine independent prognostic factors affecting progression-free survival (PFS), which subsequently informed the development of a nomogram. The performance of this prognostic model was then evaluated in the validation set using time-dependent receiver operating characteristic (ROC) curves, decision curve analysis, and calibration plots. All statistical analyses were conducted using R software, with a significance level defined as P < 0.05.

## Result

### Patient characteristics and PFS

A total of 186 patients were included in the study. The majority were male (74.2%). The median age was 44.6 years. Most patients were classified as T3 (75.8%) for tumor staging. Regarding lymph node involvement, N2 was the predominant stage (51.1%). Clinically, stage III was the most common (68.8%). For EBV levels, the group with levels ≥1500 accounted for 52.7% of patients (**Table 1**).

**Table 1:** Baseline characteristics.

Baseline characteristics of the training and validation sets	ALL	Validation cohort	Training cohort	P
	N=186	N=60	N=126	
Sex				0.995
Female	48 (25.8%)	16 (26.7%)	32 (25.4%)	
Male	138 (74.2%)	44 (73.3%)	94 (74.6%)	
Age	44.6 (10.7)	44.2 (11.8)	44.8 (10.2)	0.728
T				0.648
T2	18 (9.68%)	5 (8.33%)	13 (10.3%)	
T3	141 (75.8%)	48 (80.0%)	93 (73.8%)	
T4	27 (14.5%)	7 (11.7%)	20 (15.9%)	
N				0.991
N1	57 (30.6%)	18 (30.0%)	39 (31.0%)	
N2	95 (51.1%)	31 (51.7%)	64 (50.8%)	
N3	34 (18.3%)	11 (18.3%)	23 (18.3%)	
Stage				0.943
III	128 (68.8%)	42 (70.0%)	86 (68.3%)	
IV	58 (31.2%)	18 (30.0%)	40 (31.7%)	
EBV				0.125
<1500	88 (47.3%)	23 (38.3%)	65 (51.6%)	
≥1500	98 (52.7%)	37 (61.7%)	61 (48.4%)	

EBV, Epstein-Barr virus.

**Figure 1:**
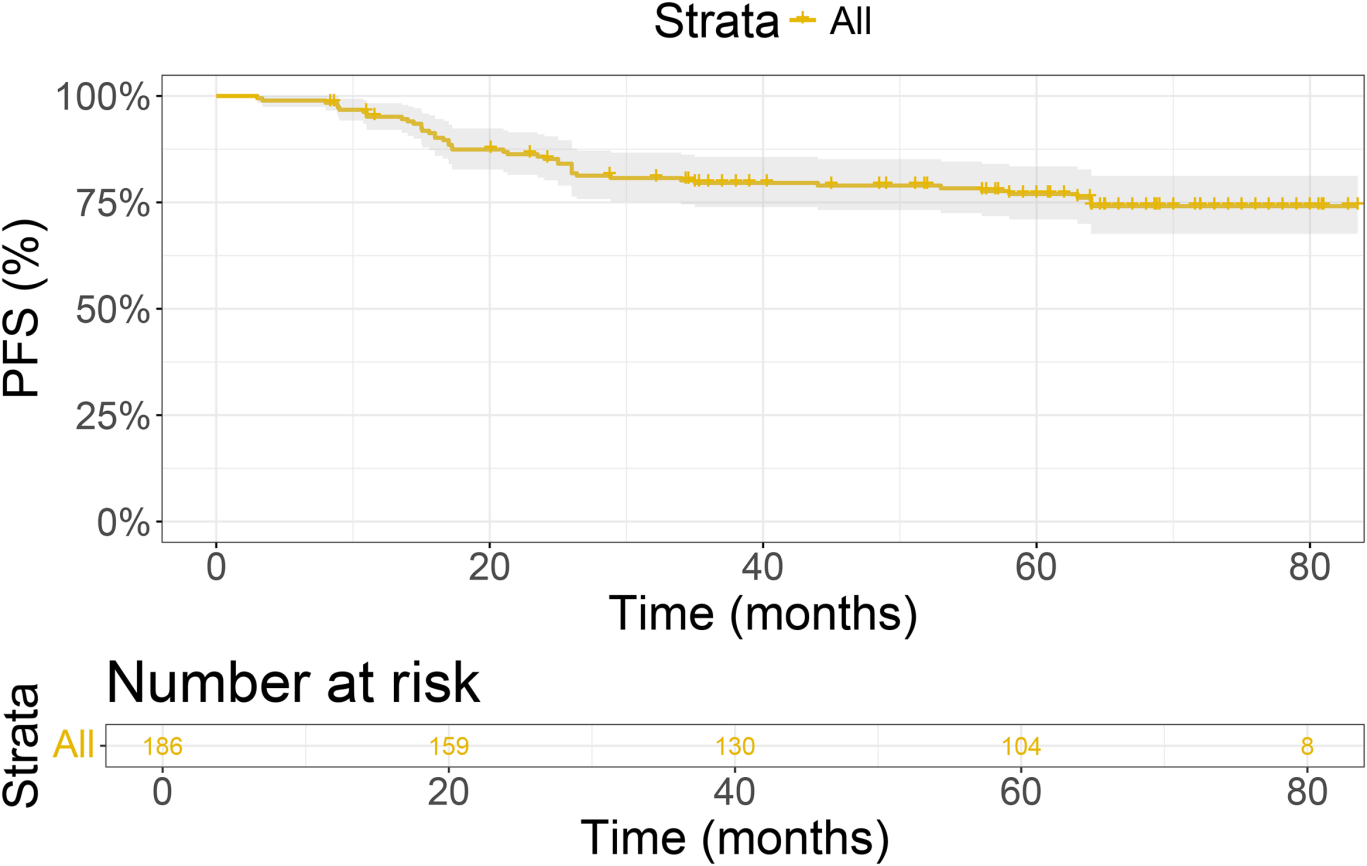
Kaplan-meier survival curve of 186 nasopharyngeal carcinoma patients.

Moreover, the 5-year PFS rate was 77% (**Figure 1**).

### Lasso+ Cox

**Figure 2:**
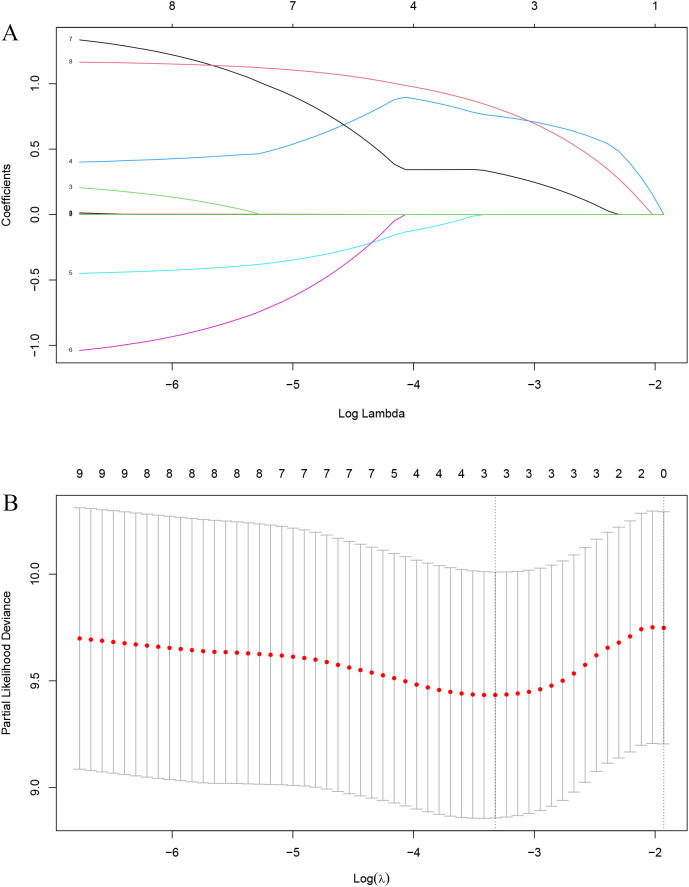
LASSO-Cox Regression Variable Selection Process. (**A**) The coefficient trajectories for variables versus log(λ). The dashed line marks the optimal λ selected via cross-validation. **(B)** The partial likelihood deviance versus log(λ), with error bars representing ±1 standard error.

All patients were randomly divided into a training set and a validation set at a 7:3 ratio, with no significant differences in baseline characteristics between the two groups (**Table 1**). Initially, LASSO regression identified three prognostic indicators: T stage, overall clinical stage, and EBV level (**Figures 2A, B**). In the training cohort, stepwise Cox regression analysis further determined that clinical stage and EBV level were independent prognostic factors for PFS (**Table 2**).

**Table 2:** Univariate and multivariate Cox regression analysis of progression-free survival.

Baseline characteristics of the training and validation sets		HR (univariable)	HR (multivariable)	HR (final)
T	T2			
	T3	1.15 (0.27-4.97, p=.854)	1.43 (0.32-6.45, p=.645)	
	T4	3.95 (0.87-17.86, p=.074)	3.11 (0.64-15.04, p=.159)	
Stage	III			
	IV	3.00 (1.46-6.17, p=.003)	1.80 (0.62-5.17, p=.278)	2.80 (1.36-5.76, p=.005)
EBV	<1500			
	≥1500	3.20 (1.46-7.00, p=.004)	3.16 (1.44-6.96, p=.004)	3.00 (1.37-6.58, p=.006)

EBV, Epstein-Barr virus.

### Nomogram

**Figure 3:**
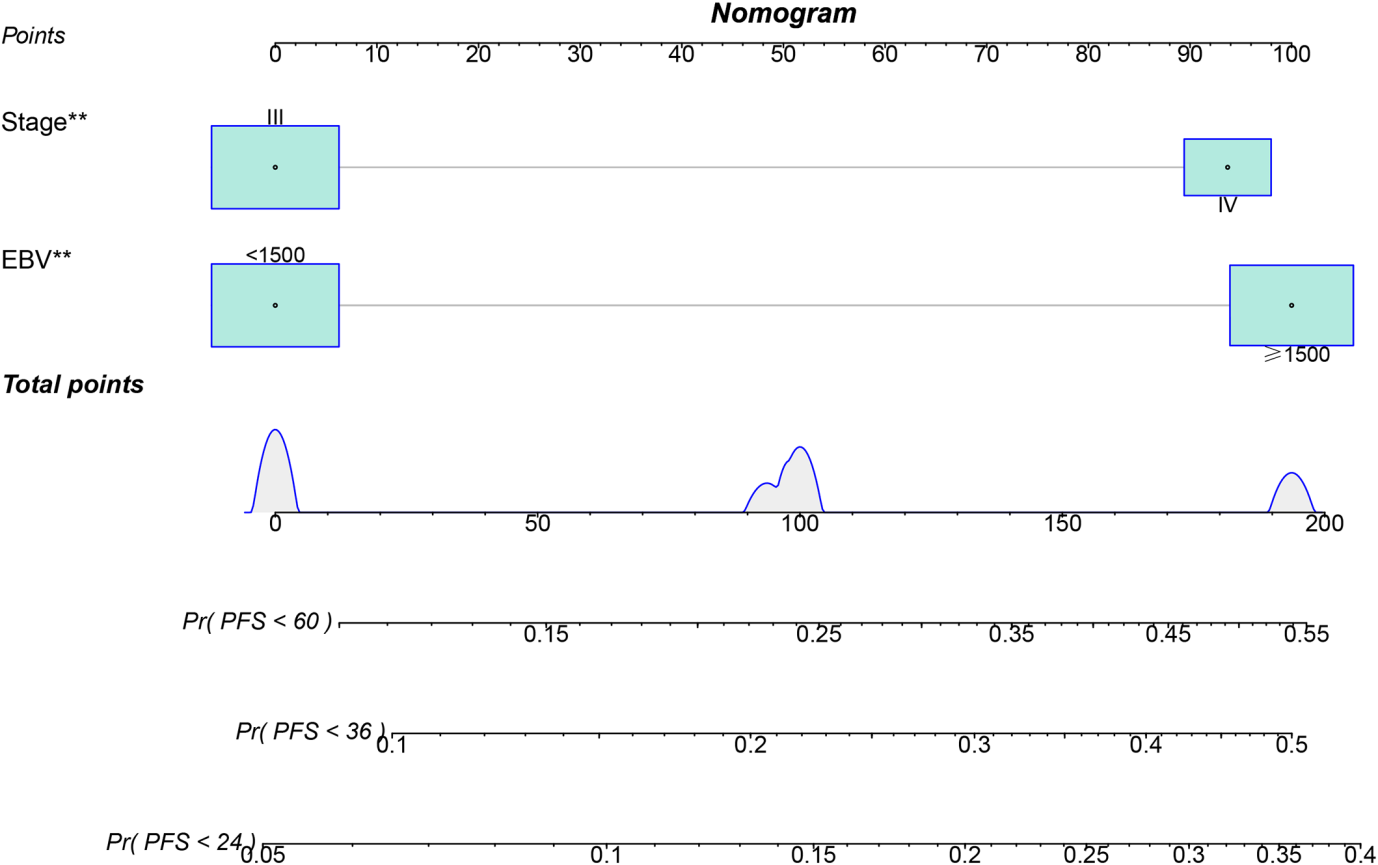
Nomogram constructed based on stage and EBV.

**Figure 4:**
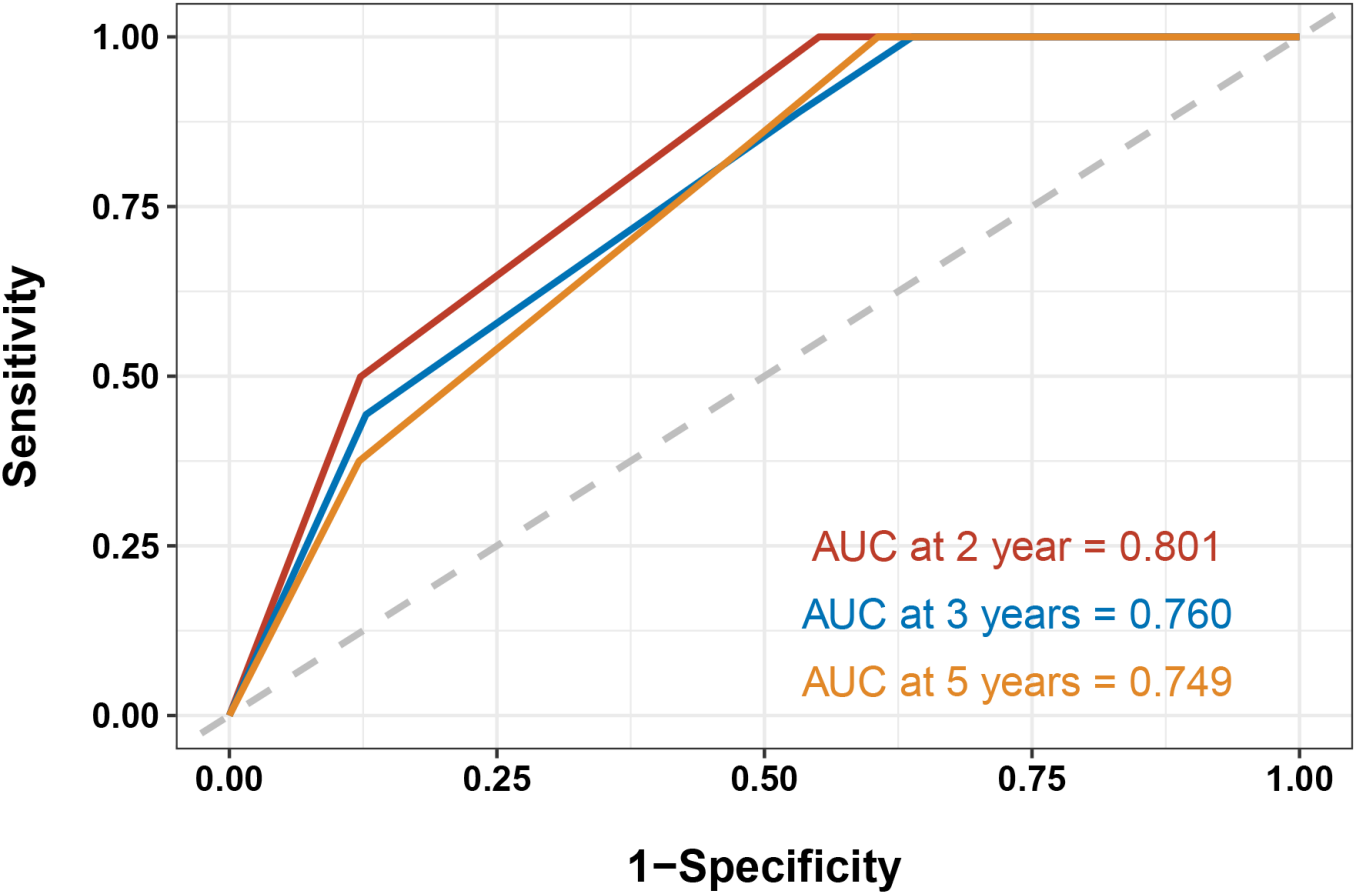
Receiver operating characteristic curves for predicting 2-, 3-, and 5-year progression-free survival in the validation cohort.

**Figure 5:**
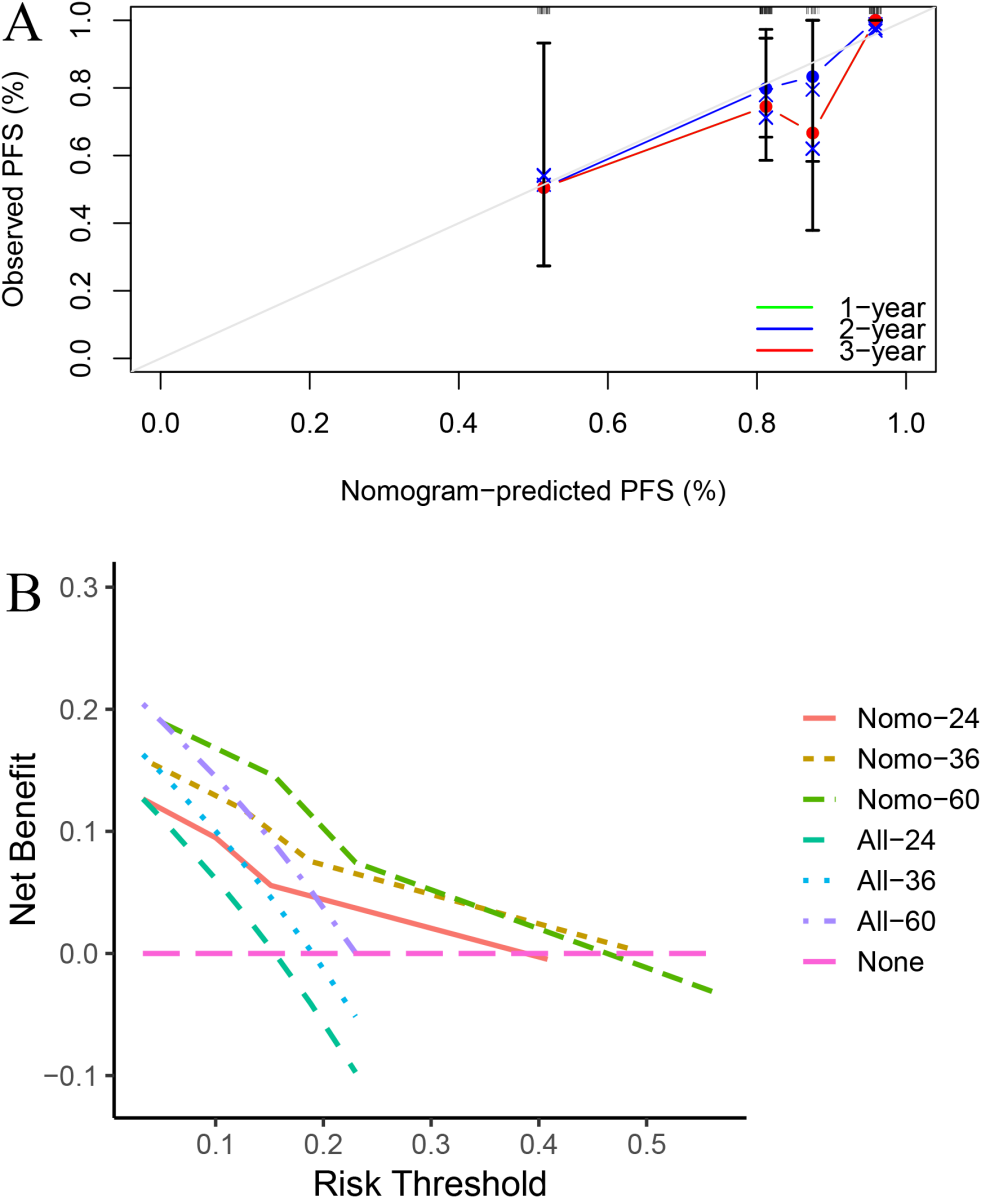
Calibration **(A)** and decision curves **(B)** confirmed that the model demonstrated robust predictive performance.

Based on these two indicators, a nomogram was subsequently developed to predict 2-, 3-, and 5-year PFS (**Figure 3**). In the validation cohort, the nomogram achieved areas under the ROC curves (AUC-ROC) values of 0.801, 0.760, and 0.749 for predicting 2-, 3-, and 5-year PFS, respectively (**Figure 4**). Calibration (**Figure 5A**) and decision curves (**Figure 5B**) confirmed that the model demonstrated robust predictive performance.

## Discuss

In this study, we developed and validated a prognostic evaluation system for patients with lymph node metastatic NPC undergoing radiochemotherapy using a LASSO-Cox regression approach. Our analysis identified clinical stage and EBV level as independent prognostic indicators for PFS. Based on these factors, a nomogram was constructed, which demonstrated robust predictive performance in both the training and validation cohorts, with AUC-ROC values of 0.801, 0.760, and 0.749 for predicting 2-, 3-, and 5-year PFS, respectively.

The significance of radiotherapy in NPC management cannot be overstated. Radiotherapy, particularly with the advent of advanced techniques such as IMRT, has revolutionized the treatment of NPC. IMRT enables precise targeting of the tumor, delivering high doses of radiation while sparing adjacent normal tissues (14). This precision is critical given the proximity of NPC to essential structures in the head and neck region. Improved local control achieved through IMRT has led to enhanced survival outcomes and reduced treatment-related toxicities (15, 16). Despite these advances, variations in individual tumor radiosensitivity, radiation dose, and fractionation schedules still contribute to heterogeneous treatment responses, emphasizing the need for reliable prognostic tools that can guide personalized treatment plans.

Our findings underscore the complementary roles of traditional clinical staging and molecular markers in prognosticating NPC outcomes. Clinical stage has long been recognized as a cornerstone in oncologic evaluation, and our study reaffirms its importance in risk stratification. In addition, EBV level, a well-established biomarker in NPC, provides valuable insights into the tumor’s biological behavior. Elevated EBV levels may indicate a higher tumor burden or more aggressive viral activity, both of which are associated with poorer clinical outcomes (17, 18). The integration of these two factors into our prognostic model not only enhances its predictive accuracy but also offers a more comprehensive understanding of the disease process in NPC (19).

In clinical applications, interpretability is essential for ensuring that predictive models are both understandable and actionable by healthcare providers. The application of the LASSO method played a crucial role in managing high-dimensional data, allowing us to select the most relevant variables while mitigating issues of multicollinearity and overfitting (20). By combining LASSO with the Cox proportional hazards model, we were able to distill complex clinical and molecular information into a concise, user-friendly nomogram (21). This tool provides a quantitative method for estimating an individual patient’s risk of disease progression over multiple time points, thereby supporting more informed and personalized treatment planning (22).

One of the advantages of using the LASSO-Cox approach lies in its ability to produce a sparse model by shrinking the coefficients of less informative variables to zero, thereby simplifying interpretation and reducing overfitting. This is particularly important in oncology, where clinicians must weigh multiple risk factors in decision-making. In our study, the LASSO method selected a minimal set of key predictors—clinical stage and EBV DNA level—both of which have strong biological relevance, making the final model more transparent and clinically meaningful.

The inclusion of EBV DNA level in our nomogram underscores its biological and clinical relevance in NPC. Epstein–Barr virus plays a pivotal role in the pathogenesis of nasopharyngeal carcinoma, particularly in endemic regions. Elevated circulating EBV DNA levels are believed to reflect higher tumor burden and are associated with increased tumor cell turnover, releasing more viral DNA into the bloodstream (23). Mechanistically, EBV can promote tumor progression by facilitating immune evasion, inducing genomic instability, and activating signaling pathways involved in cell proliferation and anti-apoptosis (24). Moreover, EBV-related oncogenesis is often accompanied by a pro-inflammatory tumor microenvironment, which may further promote tumor progression and metastasis. Clinically, pretreatment EBV DNA levels have been shown to correlate with advanced disease stage and inferior survival outcomes (25). Thus, monitoring EBV DNA not only serves as a reliable prognostic marker but also offers practical value in risk stratification, surveillance, and potentially tailoring therapy intensity—for instance, identifying high-risk patients who may benefit from escalated or adjuvant treatments.

The nomogram developed in this study offers several practical advantages. It provides clinicians with a visual tool to predict 2-, 3-, and 5-year PFS, which can be used to identify patients at high risk for disease progression. For instance, high-risk patients may be considered for more aggressive treatment strategies or closer follow-up, while low-risk patients might continue with standard therapeutic protocols, potentially avoiding unnecessary toxicity. In this way, the nomogram supports the principles of precision medicine by ensuring that treatment interventions are tailored to each patient’s unique risk profile (26).

Given that radiotherapy is the mainstay of NPC treatment, our nomogram’s ability to predict outcomes in the context of radiochemotherapy is particularly meaningful (27). The model can assist in optimizing radiation treatment plans by identifying patients who might benefit from radiochemotherapy. Such individualized approaches not only improve treatment efficacy but also contribute to better resource allocation and a reduction in adverse effects (28–33).

Our study also employed a robust validation strategy by randomly splitting the patient cohort into training and validation sets. The absence of significant baseline differences between these sets supports the generalizability of our findings. The high predictive accuracy observed in the validation cohort further reinforces the reliability of our nomogram across different patient populations and clinical settings. When compared with previous studies, our model demonstrates enhanced prognostic performance by integrating both clinical and molecular parameters, thereby bridging the gap between conventional clinical assessments and modern molecular profiling.

Despite the promising results, our study does have several limitations. First, the retrospective design introduces potential selection and information biases, as the analysis relies on historical data, which may be incomplete or inconsistent despite rigorous data standardization. Second, although our sample size is adequate for initial model development, it may not fully capture the heterogeneity of NPC observed in larger, multi-center populations. Future prospective studies involving larger cohorts are needed to further validate and refine the nomogram. Third, while our model incorporates several key clinical and molecular indicators, there remains the possibility that additional biomarkers or imaging parameters—such as genomic alterations or advanced radiologic features—could further improve prognostic accuracy. In addition, although the LASSO-Cox nomogram demonstrated strong discrimination and calibration in the internal validation cohort, these results were derived from a retrospective dataset within a limited number of institutions. As such, the generalizability of the model to broader populations and diverse clinical environments remains uncertain. Future studies should focus on validating this model in external, multicenter cohorts with varied demographic and clinical characteristics to ensure its robustness and clinical utility in real-world settings. Lastly, the selection of the regularization parameter in the LASSO method is critical; although cross-validation was used to optimize this parameter, different settings might yield slightly different results, necessitating further sensitivity analyses.

## Conclusion

In summary, our study demonstrates that a prognostic model based on the LASSO-Cox framework—integrating clinical stage and EBV level—can effectively predict progression-free survival in NPC patients undergoing radiochemotherapy. The nomogram derived from this model is a practical tool for personalized risk assessment and treatment planning. By enabling early identification of high-risk patients, our approach holds promise for guiding therapeutic decisions and ultimately improving clinical outcomes. Future research should focus on validating this model in larger, prospective cohorts and exploring the incorporation of additional prognostic markers to enhance its predictive accuracy in the era of precision oncology.

## Data Availability

The original contributions presented in the study are included in the article/supplementary material. Further inquiries can be directed to the corresponding author.
